# Can medical product development be better aligned with global needs?

**DOI:** 10.1136/bmj.o316

**Published:** 2022-02-04

**Authors:** Vasee Moorthy, Gavin Yamey

**Affiliations:** 1 Research for health department, science division, World Health Organization; 2 Center for Policy Impact in Global Health, Duke Global Health Institute, Duke University

Go to any pharmacy and you’ll see a dizzying array of shampoos and facial moisturizers with new kinds emerging all the time. There is no medical need for more ways to wash your hair or moisturize your skin, but the huge financial returns guarantee continual investment by companies into rebranding. By contrast, there are many disabling or fatal diseases for which we desperately need new medical products (such as medicines, vaccines, and diagnostics)—yet few, or none, are available and the product development pipeline is bare.^1^ ^2^ Even when these medical products do become available, often they reach high income-countries first, with a time lag of up to several decades before they become available in lower income settings. How has such inequity arisen and how can it be addressed?

## How drug companies determine whether to engage in product development

Typically, market analyses are performed by pharmaceutical companies. These analyses lead to value propositions and business cases for developing new products based on technologies those companies have either developed, or for which they have licensed intellectual property. These analyses—together with assessments of “end-user” (patient) preferences, and assessments of regulatory pathways—drive research and development (R&D) investments. In traditional for-profit product R&D, the unmet medical need is factored in only partially, including through the end-user preferences and the company’s assessment of likely regulatory authority perspectives. In some cases, governments or multilateral agencies can be large scale procurers (i.e., they will purchase the product), and in this situation their preferences may be given more weight.

However, at present, only a small proportion of global health R&D spending (around 2%) is on the compelling medical problems faced by LMICs.

One way to improve this alignment, so that product development meets the world’s medical needs, is for funders and companies to be guided by WHO target product profiles (TPPs). These profiles describe the desired characteristics of a product aimed at tackling a specific disease or diseases. These “state intended use, target populations, and other desired attributes of products, including safety and efficacy-related characteristics.”^3^

For example, in 2008, WHO developed a TPP that laid out the minimum specifications for a vaccine that would save lives by preventing a common type of pneumonia caused by *Streptococcus pneumoniae*. In 2014, WHO published a TPP for Ebola vaccines. In both cases, the needed vaccines have been developed, licensed, and provided to those most at risk following the preferences articulated by the WHO TPP documents.

These two examples show the value of TPPs. If government funders, philanthropists, and industry groups wishing to maximize the public health value of their R&D were to align their funding with WHO TPPs, they would accelerate the production and availability of technologies to curb illness and death.

WHO TPPs span the most compelling use cases for HIV, malaria, tuberculosis, and a number of neglected, vaccine-preventable and emerging infectious diseases. They are developed in all cases with a group of the world’s leading experts including scientists, product developers, public health experts, regulators, and end users from countries in different income levels.

A WHO TPP document should inform product developers, regulatory agencies, procurement agencies, and funders on R&D and public health priorities. It is intended to facilitate the most expeditious development of products addressing the greatest and most urgent public health need. WHO TPPs recognise that access, equity, and affordability are integral parts of the innovation process and need to be considered at all stages, not just after a product is developed.

## Using TPPs to guide development of vaccines for emerging infectious diseases

Novel pathogens regularly emerge from the animal-human interface, and some, such as SARS-CoV-2, will cause pandemics with incalculable social and economic cost. Yet product development in this area has been highly neglected for decades. CEPI, the Coalition for Epidemic Preparedness Innovations, receives public and philanthropic funding to advance needed vaccines for emerging infectious diseases that cause serious outbreaks. In discussions between CEPI and WHO, CEPI agreed to refer to WHO’s list of priority pathogens for investment and to advocate that vaccine development should align with WHO TPPs. In CEPI’s review of applications for funding, developers are asked to report on how their vaccine development is aligned with WHO TPP elements. In parallel, developers are encouraged to liaise with WHO early in the process to ensure that data generated will be in line with WHO’s expectations. At the same time, WHO and Gavi, the Vaccine Alliance (Gavi) hold financing discussions and WHO engages with CEPI on its access terms (to ensure that any CEPI vaccines will be accessible to low- and middle-income countries).

This end-to-end process can accelerate timelines from R&D to use by several years. A concrete example of a development direction from WHO in this area would be the focus on one dose vaccines that prevent transmission as well as disease, wherever possible. While two dose vaccines that prevent disease alone are acceptable for licensure by regulators, one dose transmission-blocking vaccines have much greater value for public health programmes. WHO has made TPPs available to guide needed covid-19 diagnostics, therapeutics, and vaccines during the ongoing pandemic.

Another example is the development of new antibiotics for highly resistant bacteria. Safe and effective antibiotics to treat otherwise incurable highly resistant, fatal bacterial infections are a compelling medical need. However, drug development in this area remains highly neglected. WHO has developed a priority list of the most compelling cases for antibiotic development, and has produced TPPs outlining minimum specifications for needed antibiotics. Ongoing horizon scanning has indicated that the existence of WHO TPPs is driving investment into the needed area, although greater investment and engagement are still needed.

## A major need for development of WHO TPPs for priority non-communicable disease indications

To date, no WHO TPPs have been developed outside infectious diseases. This is despite the fact that there are many and urgent needs for medical product development for non-communicable diseases. For example, generating data on suitability of products or devices for use in lower incomes settings can be pivotal in the pathway to use and WHO TPPs can be used to articulate these data needs. Thus, WHO TPPs can drive, not only novel product development, but also data generation that can optimize low- and middle-income country use cases for products already available in higher income settings. Certain cancers, diabetes, and hypertension all have priority indications that could serve as the basis for WHO TPP development.

## What else is essential to enable successful product development in areas where WHO TPPs call for products?

Neither push nor pull funding is likely to succeed in isolation in mobilizing funds for products identified by TPPs. Gavi and The Global Fund are clear examples of pull mechanisms. These mechanisms provide clarity to product developers that if they “build” a product in line with WHO and the global health community’s preferences, then funds will be available to procure this product. But there are major gaps in this “pull” infrastructure, notably in the areas of diagnostics and medical devices as well as therapeutics outside the remit of the Global Fund.

## Notes

Provenance and peer review: not commissioned, not peer reviewed
